# Integration of Retroviruses: A Fine Balance between Efficiency and Danger

**DOI:** 10.1371/journal.pmed.0020010

**Published:** 2005-01-25

**Authors:** Alain Fischer, Marina Cavazzana-Calvo

## Abstract

Since retroviruses can integrate a copy of their DNA into the host cell DNA, they are good vectors for gene therapy. But such vectors also have oncogenic potential

One of the key characteristics of retroviruses is their ability to integrate a copy of the DNA reverse-transcribed from their viral RNA genome into host cell DNA. This integration is mediated by a preintegration complex (PIC) comprising viral DNA, reverse transcriptase, and integrase, as well as poorly characterized host proteins [1]. It is this property that makes retroviruses good vectors for the transfer of therapeutic genes, and many such vectors have been made.

The Perspectives section is for experts to discuss the clinical practice or public health implications of a published study that is freely available online.

## Stages in Vector Development

Early vectors included those derived from murine oncoretroviruses (RVs) such as murine leukemia virus (MLV). A limitation of these viruses was that their PIC requires that the nuclear membrane is dissolved so that the PIC can come in direct contact with host cell DNA; hence, there is efficient integration only in dividing cells. Later vectors were based on lentiviruses (LVs) such as human immunodeficiency virus and simian immunodeficiency virus (SIV), whose PIC can penetrate into the nucleus so that integration can occur in nondividing cells that are in the G1 phase of the cell cycle.

## Insertional Mutagenesis

MLV-derived vectors have been used with success to achieve sustained correction of two forms of severe combined immunodeficiency (SCID)—SCID-X1 (Gamma-c deficiency) [2] and adenosine deaminase deficiency [3]. In both cases, hematopoietic progenitors were infected out of the patient's body with nonreplicative MLV-derived vectors. However, integration carries the risk of insertional mutagenesis. This mutagenesis has been demonstrated in the chicken by using replicative RVs. RV integration close to protooncogenes has been shown to induce their activation, leading to tumorigenesis. Nonreplicative MLV vectors have also been reported to induce insertional mutagenesis in a murine model [4] and, more worryingly, in two patients from the SCID-X1 trial [5]. In both instances, it is suggested that cooperation between vector-associated transgene expression (dLNGFR in one case and common Gamma chain in the other) and long terminal repeat–driven enhancement of protooncogene expression (*evl-1* and *LMO-2,* respectively) was responsible for aberrant clonal proliferation. No such events have been reported yet in the use of LV vectors in experimental settings.

## Sites of Retroviral Integration

It was initially believed that integration of retroviruses occurred randomly, but the advent of technology allowing the assessment of RV or LV integration into host cell genomes has led to a reassessment of this assumption. Using a combination of ligation-mediated polymerase chain reactions and sequencing of amplified integration sites (unique sequences made from the viral long terminal repeat and the host-genome-associated sequence), it is possible to determine all the integration sites that can be found in a given transduced cell population (or its progeny). Exact mapping can be done back to the human (or relevant animal) genome database. However, even this methodology may not detect all of the integration sites present in a given transduced cell population.

Key papers have determined the “rules of the game” for RV and LV integration into a variety of cell lines [6,7,8]; these rules seem to differ between the virus types. In both cases, however, the integration pattern is not random. RV integration tends to be close to transcription start sites of active genes—close enough to regulatory sequences to potentially exert a long terminal repeat–mediated enhancer effect [7,8]. By contrast, LVs integrate mostly in transcription units, with a preference for actively transcribed genes, but do not target the region downstream of transcription start sites [6,8]. These data indicate that there are virus-specific PIC-associated determinants that cause specific targeting with the host cell genome. However, much remains to be done to identify viral factors and host ligands involved in these interactions.

Data from these pioneering papers were obtained by in vitro infection of mature cells or cell lines with the relevant RV or LV [6,7,8]. However, it is possible that the pattern of integration might differ in other cell subsets, particularly immature cells such as hematopoietic progenitors. In fact, as shown by Mitchell et al. [8], infection of mature cells of different tissue types leads to a partially distinct pattern of integration sites related to the set of genes transcribed in these different cell types [8].

## In Vivo Models

**Figure 1:**
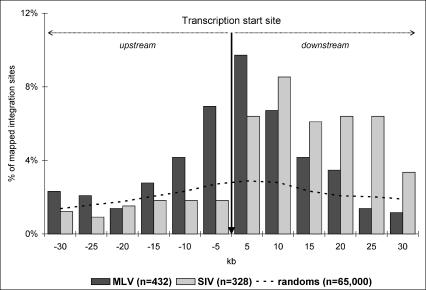
Distribution of MLV and SIV Integration Sites within a 60-kb Window Centered on Transcription Start Sites The vertical arrow points to 0 kb. Each gray bar corresponds to the percentage of SIV integration sites within a 5-kb interval, and black bars correspond to the percentages of MLV integration sites in a 5-kb interval. The distribution of a set of 65,000 in silico–generated random integration sites is represented by the dashed line. (Source: [9].)

In a paper published in last month's *PLoS Biology,* Hematti et al. [9] took the in vivo analysis further by analyzing the pattern of integration sites in cells derived from simian hematopoietic progenitor cells transduced either with a RV (MLV) or a LV (SIV) vector and transplanted into monkeys. This experimental setting is the closest possible to human trials. The results essentially confirm the nonrandom insertion pattern of both types of vectors as shown by the analysis of cell lines transduced in vitro. The analysis showed the Gaussian distribution of insertions centered on the transcription start site—thus very close to regulatory elements—of RV (Figure 1), and the preference of LV for the transcription units, with a concentration of integrations into some gene-dense regions [9]. A study such as this is therefore a useful piece of preclinical work that will help the interpretation of the analysis of clinical samples.

Similar data were also obtained by Laufs et al. in the analysis of a set of RV integration sites into human hematopoietic progenitors xenotransplanted into immunodeficient mice [10]. It thus appears that the overall distinct pattern of RV and LV integration could be independent of transduced cell types (immature versus differentiated, and tissue type). However, the targeted genes could differ considerably depending on the set of genes expressed in the target cell. Several parameters could potentially influence the transcription profile in a clinical setting, including stage of cell maturity, tissue type, ex vivo transduction culture conditions, patient age [5], underlying genetic disease, and any modification of the vector.

## Designing Future Vectors

It will thus be essential to build a free, accessible database incorporating all relevant information gathered from both experimental and clinical settings. In this respect, information gathered from the SCID-X1 and adenosine deaminase deficiency trials is awaited with great interest. Combining all available information will be the only way to determine the frequency of insertions that have a potential to induce activation of a protooncogene (a risk primarily associated with the use of RV) or to induce disruption of a regulatory gene (a risk primarily associated with the use of LV). Studies of relevant gene expression activation or suppression should therefore be carried out in parallel. It is only from these multiple analyses, including a careful comparison of the oncogenic potential of vectors in relevant animal models, that a precise assessment of the risk associated with use of retroviruses for gene therapy will come. These data will thus be the basis for objective comparison between technologies and for the design of safer vectors.

## Abbreviations

LV: lentivirus
MLV: murine leukemia virus
PIC: preintegration complex
RV: murine oncoretrovirus
SCID: severe combined immunodeficiency
SIV: simian immunodeficiency virus
